# Bioactive Metabolites from *Spilanthes acmella* Murr.

**DOI:** 10.3390/molecules14020850

**Published:** 2009-02-19

**Authors:** Supaluk Prachayasittikul, Saowapa Suphapong, Apilak Worachartcheewan, Ratana Lawung, Somsak Ruchirawat, Virapong Prachayasittikul

**Affiliations:** 1 Department of Chemistry, Faculty of Science, Srinakharinwirot University, Bangkok 10110, Thailand; 2 Department of Clinical Microbiology, Faculty of Medical Technology, Mahidol University, Bangkok 10700, Thailand; 3 Laboratory of Medicinal Chemistry, Chulabhorn Research Institute, Bangkok 10210, Thailand

**Keywords:** *Spilanthes acmella* Murr., Antioxidants, Antimicrobials, Cytotoxic effects.

## Abstract

*Spilanthes acmella* Murr. (Compositae) has been used as a traditional medicine for toothache, rheumatism and fever. Its extracts had been shown to exhibit vasorelaxant and antioxidant activities. Herein, its antimicrobial, antioxidant and cytotoxic activities were evaluated. Agar dilution method assays against 27 strains of microorganisms were performed. Results showed that fractions from the chloroform and methanol extracts inhibited the growth of many tested organisms, e.g. *Corynebacterium diphtheriae* NCTC 10356 with minimum inhibitory concentration (MIC) of 64-256 *μ*g/mL and *Bacillus subtilis* ATCC 6633 with MIC of 128-256 *μ*g/mL. The tested fractions all exhibited antioxidant properties in both DPPH and SOD assays. Potent radical scavenging activity was observed in the DPPH assay. No cytotoxic effects of the extracts against KB and HuCCA-1 cell lines were evident. Bioassay-guided isolation resulted in a diverse group of bioactive compounds such as phenolics [vanillic acid (**2**), *trans*-ferulic acid (**5**) and *trans*-isoferulic acid (**6**)], coumarin (scopoletin, **4**) and triterpenoids like 3-acetylaleuritolic acid (**1**), *β*-sitostenone (**3**), stigmasterol and stigmasteryl-3-*O*-*β*-D-glucopyranosides, in addition to a mixture of stigmasteryl-and *β*-sitosteryl-3-*O*-*β*-D-glucopyranosides. The compounds **1**–**6** represent bioactive metabolites of *S. acmella* Murr. that were never previously reported. Our findings demonstrate for the first time the potential benefits of this medicinal plant as a rich source of high therapeutic value compounds for medicines, cosmetics, supplements and as a health food.

## Introduction

*Spilanthes acmella* Murr. (Compositae) is the well known “toothache plant”, also commonly used as a spice. It has a long history of use as a folklore remedy, e.g. for toothache, rheumatism and fever [1,2]. The plant has found applications in pharmaceuticals as an antitoothache formulation for pain relief [3], swelling and gum infections [3], periodontosis [4] and in mouthwashes [5]. In addition, its extract is an active component added to body and beauty care cosmetics as a fast acting muscle relaxant to accelerate repair of functional wrinkles [6]. The plant extract was also used for stimulating, reorganizing and strengthening the collagen network in anti-age applications, e.g. in antiwrinkle cream formulations [7,8]. As a nutritional supplement [9] small amounts of the plant extract have been used for taste improvement as a sweetener with high sweetness devoid of unpleasant aftertaste that does not affect the taste or odor of foods or drinks [10].

A number of constituents had been isolated from the *S. acmella* Murr., for example, spilanthol, isobutylamides [11,12] and triterpenoids [13]. Our recent studies have shown that the *S. acmella* Murr. exhibits vasorelaxant and antioxidant activities [14]. These results motivated us to further investigate potential new compounds exerting such activities. Moreover, we have found that compounds with antioxidant action also exhibit antimicrobial activity [15]. These facts led us to search for new types of bioactive metabolites present in the *S. acmella* Murr. and examine their antimicrobial and antioxidant activities. In addition, cytotoxic effects of the plant extracts was also tested.

## Results and Discussion

### Isolation

**Figure 1:**
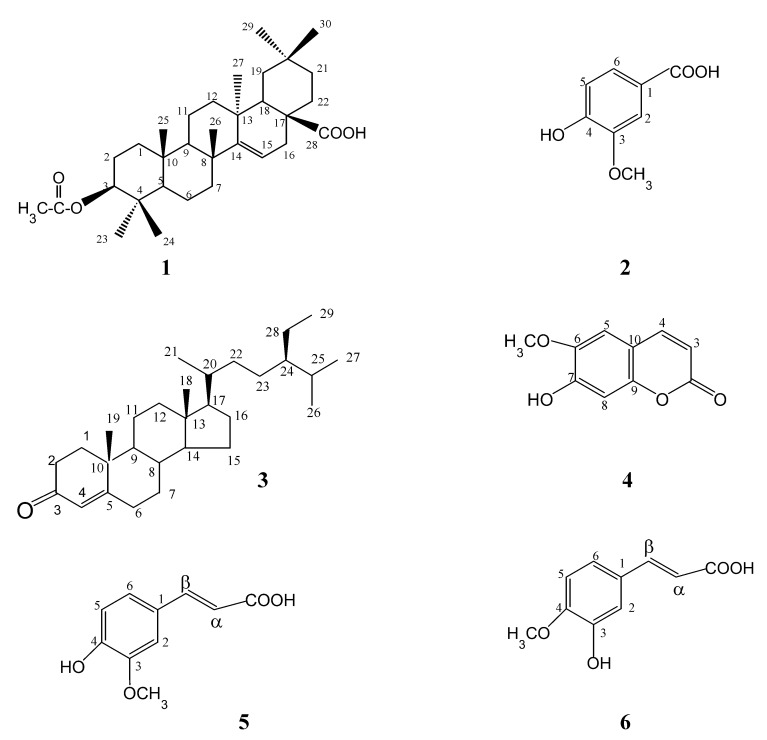
Structures of compounds **1**–**6**.

In the present study extracts, fractions and isolates of *S. acmella* Murr. were evaluated for antimicrobial, antioxidant and cytotoxic activities. Bioassay-guided isolation was carried out by repeated silica gel column using gradient elution with solvents of increasing polarity. The structures were confirmed by comparison of their spectral data (UV, IR, ^1^H- and ^13^C-NMR) with literature data. 2D NMR spectral data were also obtained. The hexane extract of *S. acmella* Murr. gave stigmasterol from fractions H1, H3, H7, while H8 including a mixture of triterpenoids. The chloroform extract provided stigmasterol from fraction C3, stigmasteryl-3-*O*-*β*-D-glucopyranoside (**SG**) from fraction C8, together with a mixture of long chain hydrocarbon esters. Fractionation of the ethyl acetate extract gave three compounds; 3-acetylaleuritolic acid (**1**), vanillic acid (**2**) and *β*-sitostenone (**3**) from fractions E5, E6, and E8, respectively. The methanol extract afforded four compounds; scopoletin (**4**), *trans*-ferulic acid (**5**), *trans*-isoferulic acid (**6**) and a mixture of stigmasteryl-3-*O*-*β*-D-glucopyranoside and *β*-sitosteryl-3-*O*-*β*-D-glucopyranoside (**MBSG**) from fractions F2, F3, M2, and M3, respectively. Isolates are summarized in Table 1 and structures of compounds **1-6** are shown in Figure 1.

**Table 1:** Isolated compounds from the fractions of the extracts.

Compound	Fraction (extract)
Stigmasterol	H1, H3, H7, and H8 (hexane)C3 (chloroform)
**SG**	C8 (chloroform)
**1, 2**, and **3**	E5, E6, and E8 (ethyl acetate)
**4**, **5**, **6**, and **MBSG**	F2, F3, M2, and M3 (methanol)

### Biological activities: Antimicrobial activity

The hexane, chloroform, ethyl acetate and methanol extracts, fractions C2-C11, C2.2, C2.3, C2.7, C3.2, E1-E14, F1-F5 and M1-M6 and isolates **1**, **2**, **4**, **5**, **6**, stigmasterol, **SG** and **MBSG** of *S. acmella* Murr. were tested for antimicrobial activity against 27 strains of microorganisms using the agar dilution method [16]. The results (Table 2) showed that hexane and chloroform extracts completely inhibited the growth of *Saccharomyces cerevisiae* ATCC 2601 with MIC 256 µg/mL. The chloroform extract also completely exhibited antigrowth activity against *Streptococcus pyogenes* II with MIC 256 µg/mL.

**Table 2:** Antimicrobial activity of *S. acmella* Murr.

Compound^a^	Organism	MIC^b^ (µg/mL)
Hexane extract	*Saccharomyces cerevisiae* ATCC 2601	256
Chloroform extract	*Saccharomyces cerevisiae* ATCC 2601	256
	*Streptococcus pyogenes* II	256
C3	*Corynebacterium diphtheriae* NCTC 10356	64
C4	*Corynebacterium diphtheriae* NCTC 10356	64
	*Bacillus subtilis* ATCC 6633	128
	*Bacillus cereus*	256
C5, C3.2, E3	*Corynebacterium diphtheriae* NCTC 10356	128
C2.2, C2.3, C2.7	*Corynebacterium diphtheriae* NCTC 10356	256
E4, E14	*Corynebacterium diphtheriae* NCTC 10356	64
M2	*Corynebacterium diphtheriae* NCTC 10356	128
	*Micrococcus lutens* ATCC 10240	128
	*Bacillus subtilis* ATCC 6633	128
	*Staphylococcus epidermidis* ATCC 12228	128
	*Bacillus* *cereus*	256
F1, F2	*Corynebacterium diphtheriae* NCTC 10356	256
	*Bacillus subtilis* ATCC 6633	128
F4, M5, M6	*Corynebacterium diphtheriae* NCTC 10356	128
	*Bacillus subtilis* ATCC 6633	128
F3, F5, M3	*Bacillus subtilis* ATCC 6633	128
M4	*Bacillus subtilis* ATCC 6633	256
Ampicillin	*Plesiomonas shigelloides*	10

a: compounds **1**, **2**, **4**, **5**, **6**, stigmasterol, **SG** and **MBSG** were tested at 64 µg/mL, no growth inhibition; b: MIC: Minimum inhibitory concentration was the lowest concentration that inhibited the growth of microorganisms.

Fractions C3, C4, C5, C2.2, C2.3, C 2.7 and C3.2 isolated from the chloroform extract exhibited antigrowth activity against *C. diphtheriae* NCTC 10356 with MIC 64-256 µg/mL. In addition, the fraction C4 also completely inhibited the growth of *B. subtilis* ATCC 6633 (MIC 128 µg/mL) and *Bacillus cereus* with MIC 256 µg/mL.

Fractions (E3, E4 and E14) of ethyl acetate extract inhibited the growth of *C. diphtheriae* NCTC 10356 with MIC 64-128 µg/mL. Antigrowth activity of ethyl acetate and methanol extracts, including fractions C2, C6-C11, E1, E2, E5-E13 and M1, were evaluated at 256 µg/mL, but no activitiy was observed. The isolates, compounds **1**, **2**, **4**, **5**, **6**, stigmasterol, **SG** and **MBSG** were tested at 64 µg/mL, but found to be inactive.

It is interesting to note that fractions from the chloroform and ethyl acetate extracts show selective growth inhibition against *C. diphtheriae* NCTC 10356 with MIC 64-256 µg/mL. Particularly, fractions C3, C4, E4 and E14 inhibited the growth of *C. diphtheriae* NCTC 10356 with MIC 64 µg/mL. All the tested methanol fractions (F1-F5, M2-M6), except M1, showed antimicrobial activity. Fractions F1-F5, M2, M3 and M5 selectively inhibited the growth of *B. subtilis* ATCC 6633 with MIC 128 µg/mL, the MIC of M4 was 256 µg/mL whereas fractions F4, M2, M5 and M6 also exhibited activity against *C. diphtheriae* NCTC 10356 with MIC 128 µg/mL. In addition, F1 and F2 exerted antigrowth activity against *C. diphtheriae* NCTC 10356 with MIC 256 µg/mL. Furthermore, M2 also inhibited the growth of *Micrococcus lutens* ATCC 10240, *Staphylococcus epidermidis* ATCC 12228 and *B. cereus* with MIC 128-256 µg/mL.

### Antioxidant activity

Fractions from the chloroform, ethyl acetate and methanol extracts were tested for antioxidant activity using the 2,2-diphenyl-1-picrylhydrazyl (DPPH) [16] and superoxide dismutase (SOD) assays [17]. The results (Table 3) showed that all the tested fractions exhibited antioxidant activity in both assays. Particularly, fractions F4, M1, M2 and M6 of the methanol extract displayed very potent antioxidant properties with 84.69-96.05% radical scavenging activity (DPPH assay), with M2 being the most potent one (96.05% activity). This led to the isolation of phenolic compound **6**. Fractions M3, M4 and M5 showed good (71.88-78.49%) antioxidant activity, whereas moderate activity was observed for F1 (48.75%) and F2 (38.29%), which yielded coumarin **4**. As for the fractions of the ethyl acetate extract, E6 exhibited the highest antioxidant activity (82.46%), while E5 and E8 showed good activity (64.75 and 76.79%, respectively). Interestingly, E6, with the highest antioxidant activity in the DPPH assay also produced the highest SOD activity (81.50%), resulting in the isolation of phenolic **2**. Triterpenoids **1** and **3** were obtained from fractions E5 and E8, respectively. Glucoside fractions **SG** (C8) and **MBSG** (M3) showed good antioxidant properties, but the stigmasterol fraction (C3) showed weak activity. However, it is noteworthy that fractions (F and M) with strong or potent antioxidants all exerted antimicrobial action too. Similar results were also found for fractions of the chloroform extract (C3-C5 including C2.2, C2.3, C2.7 and C3.2). 

**Table 3:** Antioxidant activity of *S. acmella* Murr.

Fractions^a^	Radical scavenging activity^b^ (%) (333.33 μg/mL)	NBT superoxide scavenging activity^c^ (%) (300 μg/mL)
C2	1.90	15.38
C2.2	4.78	30.94
C2.3	13.30	16.69
C2.7	6.03	19.30
C3	16.11	11.29
C3.2	6.66	28.85
C4	29.13	20.22
C5	29.01	36.31
C6	37.46	50.94
C7	50.99	34.97
C8	57.94	64.32
C9	62.51	62.22
C10	54.31	38.10
C11	73.23	20.69
E1	15.15	27.59
E3	33.45	21.27
E5	64.75	40.53
E6	82.46	81.50
E7	44.80	67.76
E8	76.79	71.20
E9	31.30	60.77
E10	36.47	57.94
E11	29.00	65.53
E12	74.05	42.29
E13	25.30	60.15
E14	39.59	52.41
F1	48.75	65.48
F2	38.29	37.28
F4	90.42	63.54
M1	84.69	50.22
M2	96.05	46.87
M3	71.88	64.72
M4	72.24	70.68
M5	78.49	58.54
M6	92.05	54.61

a: Hexane, chloroform, ethyl acetate and methanol extracts showed antioxidants (DPPH and SOD assays) [14]. b: *α*-tocopherol was used as a positive control. c: Superoxide dismutase (SOD, 3400 U/mg) from bovine erythrocytes was used as standard.

### Cytotoxic effects

The extracts of hexane, chloroform, ethyl acetate and methanol were tested against the KB and HuCCA-1 cell lines [18]. The results showed that all the extracts exhibited ED_50_ values greater than 10 µg/mL and were consequently considered to be inactive. 

Significantly, the ethyl acetate and methanol extracts displaying the most potent radical scavenging activity (DPPH) [14] afforded diverse antioxidants. There are phenolics (**2**, **5**, and **6**), coumarin (**4**), triterpenoids (**1**, **3** and **MBSG**). The most potent antioxidant fraction (SOD assay) of chloroform extract [14] afforded stigmasterol and stigmasteryl glucoside (**SG**). The former had been isolated previously from a light petrol extract of *S. acmella* Murr. along with *β*-sitosteryl-3-*O*-*β*-D-glucoside from the ethanol extract [19] and isolation of SG of the same plant had also been described [20]. Due to limited quantity of the isolates in those cases, they were not tested for antioxidants. However, all the isolates (except 3) were tested for antimicrobial activity, but no growth inhibition was observed at 64 µg/mL. The study indicates that compounds **1-6** are bioactive metabolites that have never been isolated from *S. acmella* Murr.. 

The isolated compounds had been reported to possess diverse bioactivities as follows: 3-acetyl-aleuritolic acid (**1**) had been shown to exhibit diverse bioactivities, e.g. antigrowth activity against *S. aureus* and *S. typhimurium* [21] and significant inhibition on vitality of adult male worms of *O. gutturosa* [22]. In addition, this compound showed strong inhibition of DNA topoisomerase II [23] and strong cytotoxic activity against human lung carcinoma A549 cells [23]. It had been reported that pentacyclic triterpenoids; oleanolic acid and erythrodiol exhibited vasorelaxant effect [24]. In our recent study, the observed vasorelaxant activity of *S. acmella* Murr. [14] could possibly be due to the pentacyclic, 3-acetylaleuritolic acid (**1**) isolated from fraction E5 of the ethyl acetate extract. 

Vanillic acid (**2**) had been reported to exert strong antioxidant (oral protectant) [25], powerful wound healing properties [26], protective effects against DNA damage [27] as well as antimutagenic [28] and immunostimulating [29] properties.

*β*-Sitostenone (**3**) is a triterpenoid with diverse activities such as significant hypoglycemic [30], antiarrhythmic [31] and pronounced antitubercular [32] activities. 

Scopoletin (**4**) possesses interesting activities, in particular, vasorelaxant [33], antioxidant [34], antimicrobial [35], anti-inflammatory [36], antipyretic [37], antiplatelet aggregation [38] and anti-diabetes mellitus properties [39]. In addition, it exerted neuroprotective [40] and hypotensive [41] activities in addition to applications in cardiovascular disease [39], antitumor [42], antiproliferation and antithyroid [43] treatment.

Ferulic acid (**5**) is an important natural antioxidant present in fruits, vegetables, rice bran [44], herbal medicines, beverages and supplements [45]. In addition to being an antioxidant, ferulic acid exerted a vast array of activities: e.g. vasorelaxant [46], anti-inflammatory [45], antiviral [47] and analgesic activities [48], as well as protective effects against neurodegenerative disorder (Alzheimer’s disease) [49], chemopreventive [50] and hypotensive actions [51]. Additionally, it exhibited a wide range of therapeutic effects against cancer, diabetes, cardiovascular, and neurodegenerative diseases [44]. 

Isoferulic acid (**6**) has been known as a component of Chinese herbal medicine used for a pain killer and stomachic [52]. It is a main active compound of the rhizoma of *Cimicifuga* (Japanese traditional medicine used as an anti-inflammatory [53]). 

## Conclusions

This study reports the successful isolation of a diverse group of bioactive metabolites **1-6**, stigmasterol and its glucoside together with a mixture of stigmasteryl and *β*-sitosteryl glucosides from *S. acmella* Murr.. In this and other studies these compounds possessed marked antioxidant, vasorelaxant, and antimicrobial activities including related effects, e.g. antiinflammatory, antipyretic, analgesic, antiplatelet aggregation, antidiabetic, hypotensive, neuroprotective, cardiovascular, antiviral, anticancer and chemoprotective effects. Promisingly, scopoletin (**4**) exerted antioxidant, vasorelaxant and antimicrobial actions whereas *trans*-ferulic acid (**5**) elicited antioxidant and vasorelaxant activities.

Other isolates, vanillic acid (**2**), *trans*-isoferulic acid (**6**), stigmasterol and stigmasteryl glucoside had been reported to be strong antioxidants. 3-Acetylaleuritolic acid (**1**) displayed antimicrobial and strong cytotoxic activities. *β*-Sitostenone (**3**) showed significant hypoglycemic, antiarrhythmic and antitubercular actions. These compounds **1-6** represent the bioactive metabolites that were never previously isolated from the *S. acmella* Murr.. The chloroform extract with antioxidant and antimicrobial activities afforded fractions (C3, C4, C5) of strong antigrowth actions against *C. diphtheriae* NCTC 10356 with MIC 64-128 µg/mL. Interestingly, the inactive antimicrobial extracts (ethyl acetate and methanol) provided fractions mostly with strong growth inhibition against *C. diphtheriae* NCTC 10356 and *B. subtilis* ATCC 6633 with MIC 64-128 µg/mL. Moreover, strong or potent antioxidant fractions (F, M) of methanol extract exhibited antimicrobial activity. This relation was also observed for fractions of the chloroform extract. As a result, the data support the use of *S. acmella* Murr. as a rich source of compounds with high therapeutic values for medicines, cosmetics, food supplements and as a health food.

## Experimental

### General

Melting points were determined on an Electrothermal 9100 melting point apparatus and are uncorrected. ^1^H- and ^13^C-NMR spectra were recorded on a Bruker AM 400 instrument with a 400/100 MHz operating frequency using CDCl_3_ or CD_3_OD solution with tetramethylsilane as internal standard. Mass spectra were determined using a Finnigan MAT INCOS 50 mass spectrometer. Infrared spectra (IR) were obtained on Perkin Elmer System 2000 FTIR. Ultraviolet (UV) spectra were measured with Milton Roy Spectronic 3000 Array. Column chromatography was carried out using silica gel 60 (0.063 – 0.200 mm) and silica gel 60 (<0.063 mm). Thin Layer Chromatography (TLC) and preparative TLC were carried out on silica gel 60 PF_254_ (cat. No. 7747 E., Merck).

### Plant material

Extracts (hexane, chloroform, ethyl acetate and methanol) of *S. acmella* Murr. were prepared as previously described [14].

### Cell cultures

HuCCA-1 cells were established from chlolangiocarcinomas experimentally induced in hamsters. The cell lines were characterized and have been maintained in CRI laboratory ever since 1994 in Ham’s F12 culture medium (GIBCO Laboratories, USA) supplemented with 10% fetal bovine serum (FBS, Hyclone Laboratories, USA), 100 U/mL penicillin and 100 μg/mL streptomycin. The KB cell lines, originally derived from epidermoid carcinoma of the floor of the oral cavity and commonly used as a reference laboratory standard for cytotoxicity assay, have been maintained in CRI laboratory in DMEM (Dulbecco’s modified Eagle medium).

### Isolation

Isolation was performed using conventional (gravity) column chromatography otherwise stated. A ratio of 1:30 for separated materials and silica gel was used for the chromatography. The separation was carried out using gradient elution with increasing polarity. Fractions were combined based on TLC chromatograms.

### Hexane extract

The extract (55 g) was separated to give 10 fractions (H1–H10 from hexane and dichloromethane elutions) of dark-green gum. Fractions H1, H3, H7 and H8 were rechromatographed on a silica gel column. Fraction H1 (5.31 g) gave stigmasterol (from hexane-dichloromethane elutions) 3.7 mg, m.p. 151-152 °C [54]. Fraction H3 (1.02 g) provided 7.1 mg of stigmasterol. Fraction H7 (1.34 g) gave stigmaterol 2.3 mg. Fraction H8 (8.5 g) furnished stigmasterol 1.8 mg.

### Chloroform extract

The extract (50 g) was separated to afford 11 fractions (C1–C11 from hexane-ethyl acetate and ethyl acetate-methanol elutions) of dark-green wax. Fractions C2, C3, C4, C5, and C8 were further isolated and/or purified. Fraction C2 (6.5 g) gave eight fractions (C2.1–C2.8) from chloroform-ethyl acetate elutions of the wax. Fraction C3 (5.43 g) was separated to provide six fractions of dark-green gum (C3.1–C3.6) from chloroform-ethyl acetate elutions. The fraction C3.3 (2.84 g) was further separated to give eight fractions (C3.3.1–C3.3.8) from chloroform-ethyl acetate elutions. Fraction C3.3.5 was recrystallized from methanol to give 60 mg of stigmasterol. Fraction C4 (3.65 g) was purified by column to afford 12 fractions (C4.1–C4.12 from ethyl acetate elutions). Fraction C4.1, as a dark-green solid, was recrystallized from methanol to give solid 85 mg, m.p. 61-71°C (a mixture of long chain hydrocarbon ester). Fraction C5 (3.13 g) was separated to give eight triterpene fractions (C5.1–C5.8 from ethyl acetate-methanol elutions). Fraction C8 (103.4 mg) was recrystallized from chloroform-methanol to provide solid **SG** (stigmasteryl-3-*O*-*β*-D-glucopyranoside) 15.4 mg, m.p. 261-262 °C (lit m.p. 265-267 °C [55]).

### Ethyl acetate extract

The extract (50.7 g) was applied to a silica gel column. Elution with hexane, then chloroform and ethyl acetate mixtures with increasing polarity, and finally mixtures enriched with methanol gave 14 fractions (E1 – E14). Three selected main fractions (E5, E6 and E8) were further isolated and purified. Fraction E5 (3.49 g, as a green residue from 40% ethyl acetate-chloroform elutions) was separated to obtain a yellowish green semi-solid (354.2 mg) from 40% ethyl acetate-hexane elutions. The semi-solid was purified by silica gel column chromatography. Elution with 20% ethyl acetate-hexane gave white needles which were recrystallized from methanol to give white crystals of compound **1** (3*-β-O*-acetyltaraxer-14-en-28-oic, 23.7 mg). Fraction E6 (2.78 g) obtained from 50% ethyl acetate-chloroform elutions, was further purified by column chromatography to afford an orange gum (673 mg from 50% ethyl acetate–hexane elutions). The gum was purified by column chromatography. Elution with 5% methanol-chloroform gave a white crystalline solid which was recrystallized from chloroform to give white crystals of compound **2** (4-hydroxy-3-methoxybenzoic acid, 3.8 mg). Fraction E8 (2.61 g) as a greenish gum obtained from 70% ethyl acetate-chloroform elutions, was further purified by column chromatography. Elution with 20% ethyl acetate-hexane gave a white semi-solid (27.1 mg) which was purified by repeated preparative TLC on silica gel developed 5 times with 8% ethyl acetate-hexane to give compound **3** (stigmast-4-en-3-one, 24*α*-ethyl-cholest-4-en-3-one, 4 mg, white crystals from methanol).

### Methanol extract (separated by flash column chromatography)

The extract (40.0 g) was separated on a silica gel column. Elution with chloroform then with chloroform-methanol mixtures of increasing polarity afforded five fractions (F1-F5). The main fractions F2 and F3 were further purified. Fraction F2 (3.12 g, a dark brown gum eluted by 15% methanol-chloroform) was separated to afford a brown gum (405.9 mg from 10% methanol-chloroform elutions). The gum was purified by column chromatpgraphy (elution with methanol-chloroform) to give a combined fraction (103.1 mg) which was rechromatographed on a silica gel column. Gradient elution with 3-5% methanol-chloroform gave compound **4** (7-hydroxy-6-methoxycoumarin, 3.2 mg yellowish needle-like crystals from chloroform). Fraction F3 (4.25 g) as a brown gum from 25% methanol-chloroform fractions was purified by column chromatography. Elution with 15% methanol-chloroform gave a brown semi-solid (1.02 g) which was further purified by column chromatography. Elution with 10% methanol-chloroform provided impure compound (160 mg). Recrystallization from chloroform gave compound **5** (*trans*-4-hydroxy-3-methoxycinnamic acid, 5.1 mg brownish crystals).

### Methanol extract (separated by conventional column chromatography)

The extract (123.4 g) was isolated on a silica gel column. Elution with chloroform, then gradually increasing mixtures enriched with methanol afforded six fractions (M1-M6). The main fractions M2 and M3 were purified further. Fraction M2 (642.40 mg, a greenish gum obtaining from 10-12% methanol-chloroform elutions) was separated by column chromatography. Gradient elution with 7% methanol-chloroform gave a brownish solid (30.7 mg) which was recrystallized from chloroform to give compound **6** (*trans*-3-hydroxy-4-methoxycinnamic acid, 12 mg brownish crystals). Fraction M3 (313.5 mg) as a greenish semi-solid was obtained from 20% methanol-chloroform elutions. The semi-solid (80.2 mg) was recrystallized from methanol to give compound **MBSG** (a mixture of stigmasteryl and *β*-sitostryl glucosides, 29 mg white powder). 

### Physical and spectral data

*3-Acetylaleuritolic acid* (**1**): m.p. 299-300 °C, (lit m.p. 302-304 °C [56], 304-305 °C [57]); FTIR_υ__max_ (KBr) cm^–^^1^: 3435, 2935, 1734, 1686, 1364, 1242, 1026 [57]; ^1^H-NMR (CDCl_3_) δ 5.47 (1H, *dd, J* = 3.40, 7.90 Hz, H-15), 4.39 (1H, *dd, J* = 5.50, 10.00 Hz, H-3), 1.97 (3H, *s*, COOCH_3_), 0.86 (3H, *s*, H-24), 0.81 (3H, *s*, H-27), 0.78 (3H, *s*, H-25), 1.18 (3H, *s*, H-26); ^13^C-NMR (CDCl_3_) δ 37.88 (C-4), 39.00 (C-8), 37.87 (C-10), 37.37 (C-13), 160.53 (C-14), 116.59 (C-15), 51.44 (C-17), 29.22 (C-20), 171.06 (COOCH_3_), 184.03 (COOH), 80.88 (CH-3), 55.59 (CH-5), 49.03 (CH-9), 41.58 (CH-18), 40.83 (CH_2_-7), 37.37 (CH_2_-1), 35.33 (CH_2_-19), 33.64 (CH_2_-12), 33.30 (CH_2_-21), 31.43 (CH_2_-16), 30.76 (CH_2_-22), 23.42 (CH_2_-2), 18.64 (CH_2_-6), 17.26 (CH_2_-11) 31.92 (CH_­3_-29), 28.65 (CH_­3_-30), 27.91 (CH_3_-23), 26.02 (CH_3_-26), 22.46 (CH_­3_-27), 21.21 (COOCH_­3_), 16.52 (CH_­3_-24), 15.52 (CH_­3_-25); MS *m/z* (% relative intensity): 329 (3), 269 (7), 234 (7), 189 (100) 133 (21), 119 (50) [56].

*Vanillic acid* (**2**): m.p. 210-212 °C (lit m.p. 213-214°C [58]); UV_λ__max_ (MeOH) nm (log *ε*): 253(3.41), 286(3.47) [59]; FTIR_υ__max_ cm^–^^1^: 3485, 2955, 1686, 1598, 1547, 1523, 1473, 1299, 1239, 1205, 1113, 918, 882; 819 [58];^ 1^H-NMR (CD_3_OD+CDCl_3_) δ 7.55 (1H, *d, J* = 1.90 Hz, H-2), 7.59 (1H, *dd, J* = 1.90, 8.20 Hz ,H-6), 6.88 (1H, d, *J* = 8.20 Hz, H-5), 3.92 (3H, *s,* OCH_3_); ^13^C-NMR (CD_3_OD+CDCl_3_) δ 169.11 (CO), 150.81 (C-1), 147.00 (C-3), 124.22 (C-6), 121.90 (C-4), 114.56 (C-5), 112.63 (C-2), 55.68 (OCH_3_); MS *m/z* (% relative intensity): 168 (M^+^, 100), 153 (72), 125 (35), 97 (55), 77 (5) [60].

*β-Sitostenone* (**3**): m.p. 97-99 °C, (lit m.p. 95-96 °C [61]); FTIR_υmax_ (KBr)cm^–1^: 2936, 1681, 1464, 1378, 1228 [62]; ^1^H-NMR (CDCl_3_) δ 5.74 (1H, *s*, H-4), 0.71 (3H, *s*, H-18), 0.80-1.10 (*m*, H-21, 26, 27, 29), 1.18 (3H, *s,* H-19); ^13^C-NMR (CDCl_3_) δ 29.64 (CH-25), 35.60 (CH-8), 36.07 (CH-20), 45.81 (CH-24), 53.79 (CH-9), 55.85 (CH-14), 55.99 (CH-17), 123.69 (CH-4), 11.14 (CH_­3_-29), 11.90 (CH_3_-18), 18.65 (CH_3_-19), 18.98 (CH_­3_-21), 19.75 (CH_3_-27), 20.99 (CH_­3_-26), 21.10 (CH_2_-11), 23.04 (CH_2_-28), 24.14 (CH_2_-15), 26.08 (CH_2_-23), 28.13 (CH_2_-16), 32.91(CH_2_-7), 33.86 (CH_2_-6), 33.93 (CH_2_-2), 35.65 (CH_2_-22), 36.06 (CH_2_-1), 38.57 (CH_2_-12), 39.59 (C-10), 42.35 (C-13), 171.64 (C-5),199.58 (C=O); MS *m/z* (% relative intensity): 412(M^+^,13), 397(27), 370(13), 288(26), 271(39), 229(92), 187(26), 173(46), 147(57), 124(100) [62].

*Scopoletin* (**4**): m.p. 205-206 °C (lit m.p. 203-204 °C [63]); UV_λmax_ (MeOH) nm (log ε): 294(3.68), 344(4.07) [63]; FTIR_υmax_ (KBr) cm^–1^: 3333, 1702, 1566, 1437 [64];^ 1^H-NMR (CD_3_OD) δ 6.23 (1H, *d, J* = 9.42 Hz, H-3), 7.89 (1H, *d, J* = 9.42 Hz, H-4), 6.82 (1H*, s*, H-8), 7.14 (1H*, s*, H-5), 3.91 (3H, *s,* OCH_3_), 8.10 (1H, *s*, OH); ^13^C-NMR (CD_3_OD) δ 163.00 (CO), 150.00 (C-9), 149.00 (C-7), 146.00 (C-6), 144.91 (C-4), 111.15 (C-3), 110.00 (C-10), 108.63 (C-5), 102.57 (C-8), 55.51 (OCH_3_); MS *m/z* (% relative intensity): 192 (M^+^,100), 177 (28), 164 (41), 121 (37) [65].

*trans-Ferulic acid* (**5**): m.p.168-169 °C (lit m.p. 168-169 °C [66]); UV_λmax_ (MeOH) nm (log ε): 289 (3.83), 318 (3.86) [59]; FTIR_υmax_ (KBr) cm^–1^: 3437, 1691, 1665, 1517 [54]; ^1^H-NMR (CD_3_OD) δ 7.18 (1H, *d, J* = 1.93 Hz, H-2), 7.07 (1H, *dd, J* = 1.93, 8.23 Hz, H-6), 6.82 (1H, *d, J* = 8.23 Hz, H-5), 6.31 (1H, *d, J* = 15.88 Hz, H-*α*), 7.59 (1H, *d*, *J* = 15.88 Hz, H-*β*), 3.89 (3H*, s,* OCH_3_); ^13^C-NMR (CD_3_OD) *δ* 171.19 (CO), 151.50 (C-3), 149.90 (C-4), 127.76 (C-1), 123.97 (C-6), 116.46 (C-5), 115.89 (C-*α*), 111.64 (C-2), 146.95 (C-*β*), 56.45 (OCH_3_); MS *m/z* (% relative intensity): 194 (M^+^,100), 179 (16), 161 (5), 148 (6), 133 (17), 105 (5), 77 (6).

*trans-Isoferulic acid* (**6**): m.p. 230-232 °C (lit m.p. 230 °C [66]); UV_λmax _(MeOH) nm (log ε): 289 (3.93), 313 (3.97) [59]; FTIR_υmax_ (KBr) cm^–1^: 3437, 2968, 1692, 1665, 1620, 1600, 1517, 1277, 1206, 1178 [54]; ^1^H-NMR (CD_3_OD+CDCl_3_) *δ* 6.26 (1H, *d, J* = 15.90 Hz, H-*α*), 7.61 (1H, *d, J* = 15.90 Hz, H-*β*), 7.07 (1H, *d, J* = 1.67 Hz, H-2), 7.05 (1H, *dd, J* = 8.00, 1.67 Hz, H-6), 6.87 (1H, *d, J* = 8.00 Hz, H-5), 3.91 (3H, *s*, OCH_3_);^13^C-NMR (CD_3_OD+CDCl_3_) *δ* 110.50 (CH-2), 115.28 (CH-*α*), 115.57 (CH-5), 123.28 (CH-6), 126.83 (C-1), 146.11 (CH-*β*), 170.25 (CO), 148.98 (C-3), 147.97 (C-4), 56.04 (OCH_3_); MS *m/z* (% relative intensity): 194 (M^+^,100), 193 (28), 179 (23), 177 (12), 148 (6), 133 (28), 105 (14), 77 (12).

*Mixture of stigmasteryl-3-O-β-D-glucopyranoside and* *β-sitosteryl-3-O-β-D-glucopyranoside* (**MBSG**): m.p. 261-262 °C (lit m.p. 264-266 °C [55], 278-290 °C [67]); FTIR_υmax_ (KBr) cm^–1^ : 3406, 2935, 1654, 1459, 1368, 1024; ^1^H-NMR (CDCl_3_+CD_3_OD) *δ* 0.64-2.50 (*m*,CH, CH_2_,CH_3_ of steroid) 3.20- 3.40, 3.72-3.88 (m, glucosidic protons), 3.56-3.64 (*m*, 1H, H-3), 4.42 (1H, *d, J* = 7.83 Hz, H-*β-*anomeric), 5.03(1H, *dd*, *J* = 15.66, 8.75 Hz, H-22^+^), 5.17 (1H, *dd*, *J* = 15.63, 8.62 Hz, 23^+^), 5.38 (1H, *t*, *J* = 3.59, H-6); ^13^C-NMR (CDCl_3_+CD_3_OD) *δ* 69.52 (CH-3), 121.93 (CH-6), 49.94 (CH-9), 31.76 (CH-8), 56.61 (CH-14), 55.81 (CH-17), 40.28 (CH-20), 138.11 (CH-22^+^), 129.05 (CH-23^+^), 51.02 (CH-24), 33.69 (CH-25), 11.76 (CH_3_-18), 19.51(CH_3_-19), 20.93 (CH_­3_-21), 19.02 (CH_­3_-26), 20.80 (CH_3_-27), 11.95 (CH_3_-29), 36.99 (CH_2_-1), 29.30 (CH_2_-2), 41.97 (CH_2_-4), 31.63 (CH_2_-7), 22.81 (CH_2_-11), 39.51 (CH_2_-12), 24.04 (CH_2_-15), 28.89 (CH_2_-16), 33.69 (CH_2_-22^++^), 27.99 (CH_2_-23^++^), 25.17 (CH_2_-28), 140.05 (C-5), 36.47 (C-10), 42.08 (C-13), 100.84 (C-1'), 73.26 (C-2'), 76.14 (C-3'), 69.52 (C-4'), 76.67 (C-5'), 61.22 (C-6'); MS *m/z* (% relative intensity): 414 (6), 412 (7), 393 (57), 394 (84), 381 (33), 300 (75), 287 (66), 255 (100), 227 (37), 213 (61), 147 (87), 145 (90), 131 (49), 105 (66), 91 (87), 79 (44); ^+^ is ^1^H-NMR and ^13^C-NMR of stigmasteryl-3-*O*-*β*-D-glucopyranoside, ^++^ is ^13^H-NMR of *β*-sitosteryl-3-*O*-*β*-D-glucopyranoside.

### Biological evaluations

*Antimicrobial assay:* Antimicrobial activity of the plant extracts, fractions and isolates (**1**, **2**, **4**, **5**, **6**, stigmasterol, **SG** and **MBSG**) was investigated using the agar dilution method [16]. Briefly, the test compounds dissolved in either CH_2_Cl_2_ or MeOH were individually mixed with Müller Hinton (MH) broth to obtain a final volume of 2 mL. A two-fold dilution was prepared and the solution was then transferred to the MH agar solution to yield the final concentrations ranging from 4-256 μg/mL. Twenty seven strains of microorganisms (Table 4), cultured in MH broth at 37 °C for 24 h, were diluted with 0.9 % normal saline solution to adjust the cell density of 10^8^ CFU/mL. The organisms were inoculated onto each plate using a multipoint inoculator and further incubated at 37 °C for 24-48 h. Compounds which possessed high efficacy to inhibit bacterial cell growth were analyzed.

**Table 4:** The twenty-seven strains of microorganisms used for antimicrobial activity testing.

	Reference strains	Clinical isolates
Gram-negative bacteria	*Escherichia coli* ATCC 25922	*Shigella dysenteriae*
	*Klebsiella pneumoniae* ATCC 700603	*Salmonella enteritidis* type C
	*Serratia marcescens* ATCC 8100	*Morganella morganii*
	*Salmonella typhimurium* ATCC 13311	*Aeromonas hydrophila*
	*Shewanella putrefaciens* ATCC 8671	*Citrobacter freundii*
	*Achromobacter xylosoxidans* ATCC 2706	*Plesiomonas shigelloides*
	*Pseudomonas aeruginosa* ATCC 15442	
	*Pseudomonas stutzeri* ATCC 17587	
Gram-positive bacteria	*Staphylococcus aureus* ATCC 29213	*Streptococcus pyogenes* II
	*Staphylococcus aureus* ATCC 25923	*Bacillus cereus*
	*Staphylococcus epidermidis* ATCC 12228	*Listeria monocytogenes*
	*Enterococcus faecalis* ATCC 29212	
	*Enterococcus faecalis* ATCC 33186	
	*Micrococcus lutens* ATCC 10240	
	*Bacillus subtilis* ATCC 6633	
	*Corynebacterium diphtheriae* NCTC 10356	
Yeasts	*Saccharomyces cerevisiae* ATCC 2601	
	*Candida albicans* ATCC 90028	

*Antioxidative assay*: The antioxidative activity of the extracts was elucidated by the DPPH radical scavenging assay [16]. Experiments were initiated by preparing a 0.1 mM solution of DPPH in methanol. One mL of this solution was added to a sample solution (0.5 mL, 1 mg/mL dissolved in methanol). After 30 min, absorbance at 517 nm was measured and the percentage of radical scavenging activity was calculated from the following equation:

% Radical scavenging = (1-Abs.sample/Abs.cont)×100

where Abs.cont is the absorbance of the control reaction and Abs.sample is the absorbance of the tested sample. The SOD activity was assayed by measuring inhibition of the photoreduction of nitro blue tetrazolium (NBT) [17]. The indirect assay is comprised of several reactions: the photochemically excited riboflavin was first reduced by methionine into a semiquinone, which donated an electron to oxygen to form a superoxide source. The superoxide readily converted NBT into a purple formazan product. As a result, the SOD activity was inversely related to the amount of formazan formed.

*Cytotoxic assay*: Cytotoxic activity of the plant extracts was determined by a slightly modified method described previously [18]. Briefly, the confluent cell monolayers were trypsinized and diluted with appropriate culture medium to a final concentration of 3×10^5 ^cells/mL. Portions (100 μL) containing approximately 3×10^4^ cells were distributed into 96-well flat-bottomed tissue culture plates and incubated overnight at 37^°^C in a humidified 5% CO_2_ incubator. Solutions (100 μL) containing different concentrations of tested extracts (0.001–10 μg/mL) or taxol (0.012–1.2 μg/mL) were added to each well and the plates were incubated as above for an additional 48 h. After the incubation, each well was washed (x 3) with phosphate-buffered saline (pH 7.2) and then stained with Crystal Violet. After the excess dye was removed, the stained cells were lysed with 100 mM HCl (100 μL) in absolute methanol and the optical density was determined by a microtitre plate reader (Titertek, Multiskan MCC/340) set to read at a wavelength of 540 nm. All tests were carried out in quadruplicate and the mean value was calculated. The activity was expressed as ED_50_ (the effective dose that inhibits 50% of cell growth).
